# Compressed Air, Its Uses in Dentistry

**Published:** 1907-08-15

**Authors:** W. F. Linnert


### COMPRESSED AIR, ITS USES IN DENTISTRY.

BY W. F. LINNERT, D.D.S.

Pressure ranging from 20 to 30 pounds I find most preferable. This amount of pressure gives a good strong stream of air and not too strong, have a fairly long nozzle about, six inches is long enough, having a small hole and reinforced at opening, so when a hot blast is required it may be heated and retain its heat for a few moments, next have a good air valve. The best is (the Globe), working on the same principal as our common gas cut off, being tapered, it will always remain tight, then last but not least have a large air tank, the larger the better, however a small one will do by pumping oftener. The following are its uses:

First. In making an examination, in locating cavities, you can use it in blowing out any substance that may hide in any small cavity and if your mirror fogs simply blow a little air on same and in an instant it is clear again and you may continue your examinations.

Second. In drying cavities use warm air and carefully control the flow of air and you will soon appreciate its usefulness in this operation.

Third. In treating putrescent teeth you will find it more effective than anything you have or can do. We all know what it means when a patient comes in saying, Doctor, my tooth is an inch or a foot, or some even get it so enlarged that they make it a yard longer than the rest and heat hurts it, etc. When opening this tooth a fragrant odor greets us, announcing the death of the pulp, here with your hot compressed air blasts, after removing as much of the putrescent matter as possible and having flooded cavity with alcohol you thoroughly dry this tooth each root up to its apex and success is certainly yours 99 times out of a 100 cases.

Fourth. In crown setting, to dry the tooth to be crowned which should be thoroughly dry, and be done instantly with a good heavy stream of air.

Fifth. In bridge setting, drying the abutments, its use is far surpassed from the old hand bulb air syringe.

Sixth. In sensitive dentine, after applying your carbolic acid or other remedies, such as chloroform, ether and menthol or whatsoever you may use, into the cavity to be excavated on a pledget of cotton you can evaporate the dentine by a continual warm stream of air and will find results most favorable; a trial will convince you.

Seventh. In taking an impression when the patient is easily gaged, herein using 1 per cent solution cocain in an atomizer and thoroughly spray the palate you will find it favorable.

Eigth. In drying cement fillings and polishing gold fillings with a rapid moving disk you will generally heat, here compressed air, a steady stream will save time as well as pain to the patient.

Ninth. In inlay work, when absolute dryness is required until cement is thoroughly set, compressed air will save at least one-third of your time, for you can dry the cement with a constant blast from your nozzle, so that even the dampness from the breath will have any effect.

Tenth. In your laboratory you will find compressed air a good companion in using your blow pipe. Set aside your bellows, be steady and get a steady flame and be at ease yoursel,f having no bellows on which to pump around on. With these few remarks I shall bring this subject to a close; having gone over it as brief as possible—perhaps too much so. If so I am ever ready to give any information desired and will gladly demonstrate its uses to any one.

I only hope that each and every one will give this subject a thorough investigation and I am sure if used once by anyone of our noble profession it will never be discontinued for it is certainly a thorough dryer and a time saver, which means success to the very busy dentist and dollars in his pocket. For time is money.—*Dental Era.*

